# *Tomosaic*: efficient acquisition and reconstruction of teravoxel tomography data using limited-size synchrotron X-ray beams

**DOI:** 10.1107/S1600577518010093

**Published:** 2018-08-21

**Authors:** Rafael Vescovi, Ming Du, Vincent de Andrade, William Scullin, Doǧa Gürsoy, Chris Jacobsen

**Affiliations:** a Advanced Photon Source, Argonne National Laboratory, Argonne, IL 60439, USA; b Department of Materials Science, Northwestern University, Evanston, IL 60208, USA; c Argonne Leadership Computing Facility, Argonne National Laboratory, Argonne, IL 60439, USA; d Department of Electrical Engineering and Computer Science, Northwestern University, Evanston, IL 60208, USA; e Department of Physics and Astronomy, Northwestern University, Evanston, IL 60208, USA; f Chemistry of Life Processes Institute, Northwestern University, Evanston, IL 60208, USA

**Keywords:** X-ray tomography, image alignment, image reconstruction, parallelized computing

## Abstract

X-rays offer high penetration with the potential for tomography of centimetre-sized specimens, but synchrotron beamlines often provide illumination that is only millimetres wide. Here an approach is demonstrated termed *Tomosaic* for tomographic imaging of large samples that extend beyond the illumination field of view of an X-ray imaging system. This includes software modules for image stitching and calibration, while making use of existing modules available in other packages for alignment and reconstruction. The approach is compatible with conventional beamline hardware, while providing a dose-efficient method of data acquisition. By using parallelization on a distributed computing system, it provides a solution for handling teravoxel-sized or larger datasets that cannot be processed on a single workstation in a reasonable time. Using experimental data, the package is shown to provide good quality three-dimensional reconstruction for centimetre-sized samples with sub-micrometre pixel size.

## Abstract 2

An approach is demonstrated termed *Tomosaic* for tomographic imaging of large samples that extend beyond the illumination field of view of an X-ray imaging system.

## 1. Introduction   

Computed tomography (CT) allows one to obtain internal structure of a three-dimensional sample from of a series of two-dimensional projection images collected around a common rotation axis. When using X-rays rather than visible-light or electron microscopy, CT is especially powerful because of the ability to image centimetre-sized or larger objects (Stock, 2008*a* ▸). Illuminating centimetre-sized objects is straightforward when using cone-beam illumination from laboratory-based electron-impact sources which emit into a solid angle approaching π; however, with laboratory-based systems it becomes challenging to obtain both submicrometre voxel resolution and centimetre-sized fields of view in reasonable experimental times. If one instead uses a synchrotron radiation source for its higher spectral flux and its parallel-beam geometry, relativistic effects limit the angular extent of the beam so that even at the 20–50 m distance of many experimental enclosures from the X-ray source one often has a beam that is at most a millimetre or two in width (Weitkamp *et al.*, 2010 ▸). While there are a limited number of long wiggler-source beamlines that can provide illumination over much larger specimen widths (Nemoz *et al.*, 2007 ▸), they deliver a lower photon density on the specimen so that they are less well suited for micrometre-resolution studies. Therefore there is a need for a method for imaging centimetre-sized samples at sub-micrometre resolution using millimetre-sized beams at the synchrotron light sources of today.

Consider a realistic example of three-dimensional imaging of a 2 cm-sized specimen with 1 µm resolution [a spatial resolution that is achievable using a scintillator imaged with a microscope objective onto a visible-light camera (Flannery *et al.*, 1987 ▸)]. With such a sample, one would like to acquire projection images with 20000 pixels on a side. Not only is it difficult to illuminate such an imaging field for the reasons described above, it is also difficult to obtain a high signal-to-noise ratio (SNR) small-pixel-size visible-light camera with such a large number of pixels in a single device.

**Figure 1:**
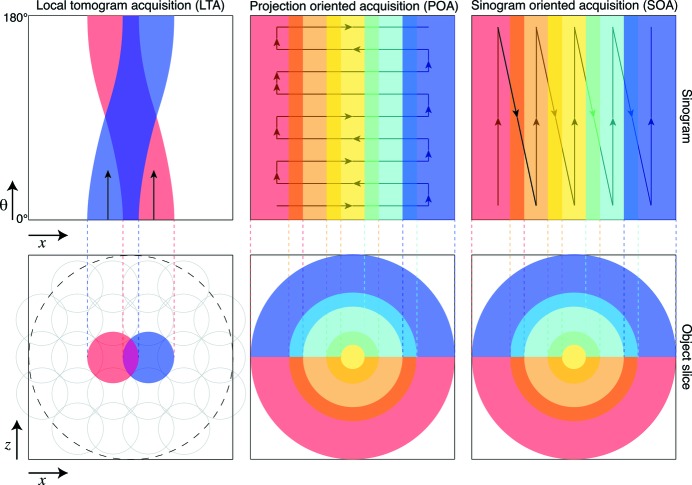
Schematic comparison of three methods for X-ray computed tomography of specimens that are larger than the illumination footprint and detector size, as viewed from above for one object slice if the rotation axis is vertical. The bottom figure row shows views of one slice within the object corresponding to one detector row, while the top figure row shows sinograms from one detector row as the object is rotated. In the local tomography acquisition (LTA) or interior tomography *Tomosaic* approach, the rotation center is placed within a subregion of the object after which a rotation sequence is acquired; when shown in the overall context of the object, one therefore obtains the corresponding sinogram above. In a projection-oriented acquisition (POA) approach as shown in the middle, one acquires a tiled set of projection images while the object is moved transverse to the illumination direction; the object is then rotated slightly about its overall center and the sequence repeated. In the sinogram-oriented acquisition (SOA) *Tomosaic* approach shown on the right, the rotation center is moved to an offset position relative to the illumination and detector, and the object is rotated to acquire data from a ring-within-a-cylinder region. Both the POA and SOA *Tomosaic* approaches involve less exposure overlap, reducing radiation dose. Furthermore, SOA generally provides faster acquisition speed because it involves fewer translational motions from the sample stage.

In order to obtain tomographic reconstructions of an object that is larger than the field of view of the illuminating beam and the detector without sacrificing spatial resolution, several approaches have been described previously (Kyrieleis *et al.*, 2009 ▸); we describe three main ones here: a local tomography acquisition approach, a projection-oriented acquisition approach, and a sinogram-oriented acquisition approach. These acquisition methods (which are illustrated in Fig. 1 ▸, and summarized in Table 1 ▸) have the following characteristics:

(i) Local tomography acquisition (LTA). One approach is to acquire and reconstruct a series of local tomograms of subregions of the specimen by successively placing each subregion on the rotation axis (Kuchment *et al.*, 1995 ▸; Oikonomidis *et al.*, 2017 ▸) [method III of Kyrieleis *et al.* (2009 ▸)], as shown in Algorithm 1. This is also known as truncated object tomography (Lewitt & Bates, 1978 ▸), or as interior tomography (Natterer, 1986 ▸). In this scheme, features from outside the reconstructed region are present in only a small subset of the acquired projections. Therefore they contribute only weakly to the local reconstruction volume, though they do introduce some imaging artifacts (Kyrieleis *et al.*, 2011 ▸). After reconstruction, these local reconstructed tomogram volumes are stitched together to reconstruct the full three-dimensional volume.
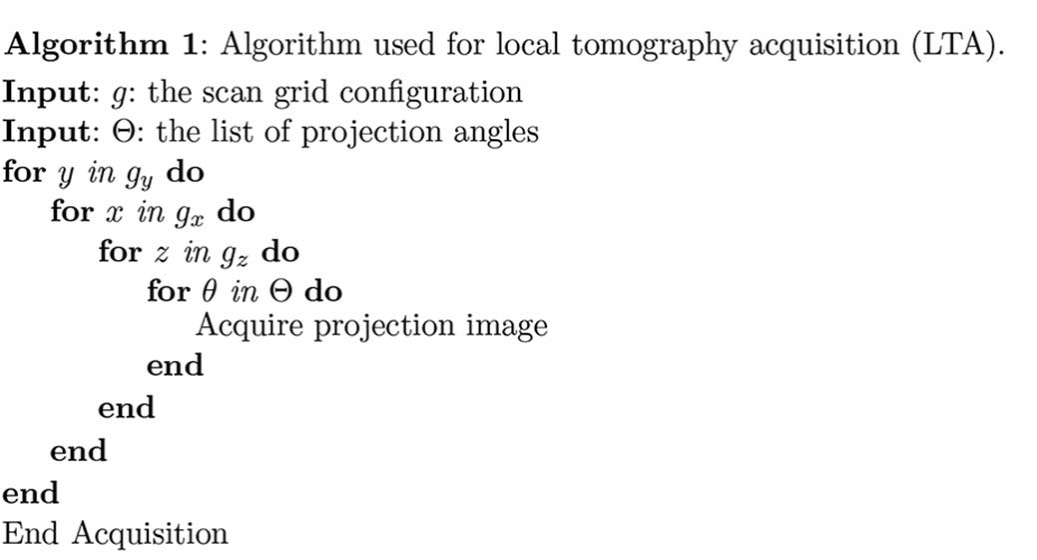


(ii) Projection-oriented acquisition (POA). In this approach, one collects a mosaic tiling of two-dimensional images at each projection angle θ (Algorithm 2), after which these images are stitched together to create a single two-dimensional projection for that angle. These projections can then be stacked in angle to create a sinogram of the full three-dimensional volume, after which a tomographic reconstruction is obtained. This approach [method I of Kyrieleis *et al.* (2009 ▸)] has been used for example with Fresnel zone plate optics for sub-100 nm-resolution tomography (Liu *et al.*, 2012 ▸; Mokso *et al.*, 2012 ▸).
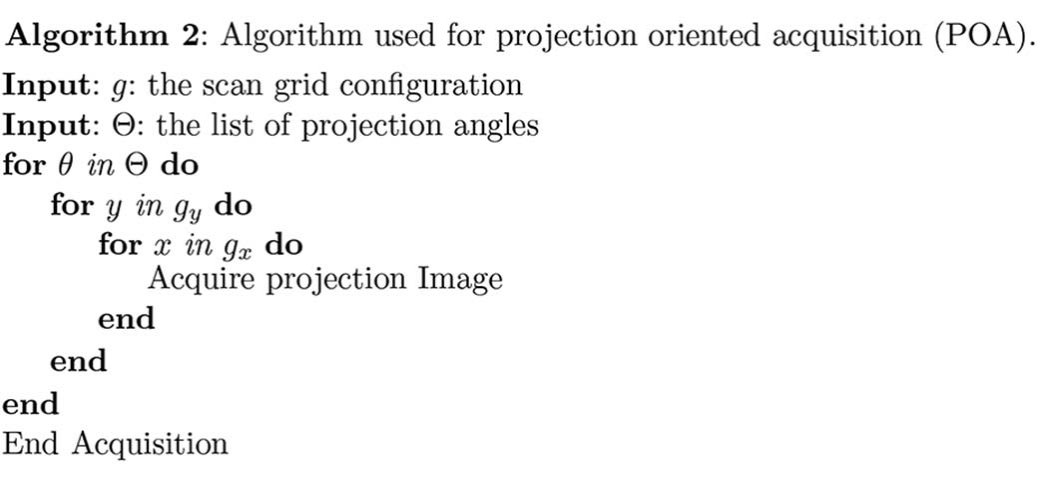


(iii) Sinogram-oriented acquisition (SOA). In this approach, one acquires data using a fixed horizontal and vertical or 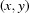 offset between the field of view and the specimen rotation axis. One then moves to the next 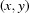 offset before another rotation dataset is acquired (Algorithm 3) (Vescovi *et al.*, 2017 ▸). In this way [method V of Kyrieleis *et al.* (2009 ▸)], each rotation series provides a subregion of the full three-dimensional sinogram which is complete in θ and incomplete in *x*. These ‘ring in a cylinder’ projection sets must then be aligned and assembled prior to reconstruction of the full three-dimensional volume (Vescovi *et al.*, 2017 ▸).
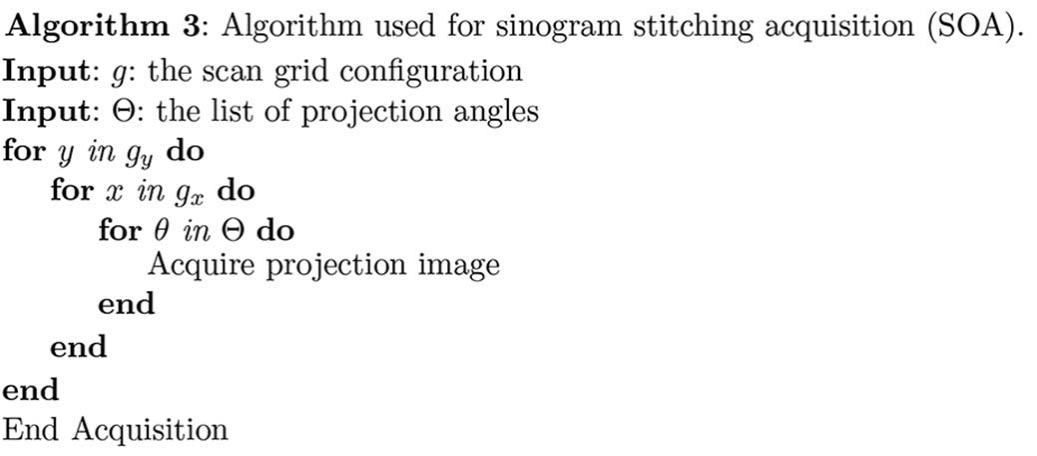


While reconstruction of samples with up to two times larger size than the illumination and detection field of view can be achieved by placing the rotation axis on the illumination/detection boundary (Stock, 2008*b* ▸) [method II of Kyrieleis *et al.* (2009 ▸)], this technique does not scale to larger volumes.

The three approaches described above each make different tradeoffs in data acquisition and processing. To describe this, we use a geometry shown in Figs. 1 ▸ and 3 where the object is rotated about the *y* axis (vertical in the case of our X-ray tomography setup), and reconstructed tomographic slices are in the *x*–*z* plane. Consider the case where each field of view contains 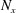 and 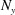 pixels in the *x*- and *y*-direction, respectively. Ignoring overlaps between fields of view, the total number of voxels in the reconstructed object using any of LTA, POA and SOA will be given by
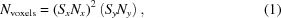
where 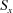 and 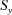 represent the number of fields of view along the *x*-direction (horizontal and perpendicular to the beam) and *y*-direction (vertical), respectively. For LTA, there are multiple scan regions also along the *z*-direction (parallel to the beam), but we assume 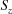 = 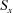 for a roughly square-shaped sample. We then notice the following differences among LTA, POA and SOA:

(i) In LTA, one needs to work with a data size of 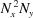 for each reconstruction, so that tomogram reconstruction software optimized for one illumination/camera field of view can be used unaltered. In addition, one can examine each local tomogram region as it is acquired, without waiting for the entire specimen’s data to be collected. However, one must then assemble these 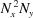 size reconstruction volumes into an overall volume of 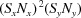. In both the POA and SOA approaches, one must reconstruct the complete 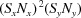-sized dataset before any subregion of the reconstructed volume can be viewed, which can lead to computational challenges as described below.

(ii) In the LTA approach, most regions of the specimen are exposed to the beam more than once, as can be seen in the sinogram representation of Fig. 1 ▸. This can increase the radiation dose to the specimen, unless approaches analogous to dose fractionation are used (Hegerl & Hoppe, 1976 ▸). Assuming that the sample is square in shape, LTA requires a larger number of 180° scans than POA and SOA by a factor of 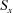 if a square grid on the *xz*-plane is used, or 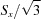 if a hexagonal grid is used. These tradeoffs are investigated in more detail in simulation studies (Du *et al.*, 2018 ▸).

(iii) The specimen stage motions between these approaches are quite different. In the LTA approach, one must translate selected specimen 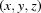 positions onto the rotation axis, and then acquire a rotation angle dataset. In POA, one translates the specimen in 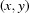 at each angle θ, after which the translation sequence must be repeated. In the SOA approach, one translates the rotation stage in 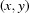, and then acquires a complete rotation angle dataset at that 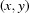 position. In practice, high-precision rotation stages can quickly rotate a specimen over 180°, whereas translations in 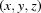 tend to take a longer time to allow for acceleration, deceleration and settling at the end position, so that the SOA approach is favored.

As can be seen from the above, POA and SOA offer advantages compared with LTA in terms of dose efficiency. This is a crucial factor to be considered when imaging soft materials. With today’s computing resources and parallel computation techniques, the computational burden of POA and SOA can be overcome, as will be shown in the *Results* [Sec sec4] section. Furthermore, when comparing POA and SOA one will notice that fewer translational motions are needed by SOA, which makes SOA a faster acquisition method than POA. We thus consider SOA to be the optimal tomographic acquisition method for large radiation-sensitive samples. In the experiments described below, we have used the SOA approach.

**Figure 2:**
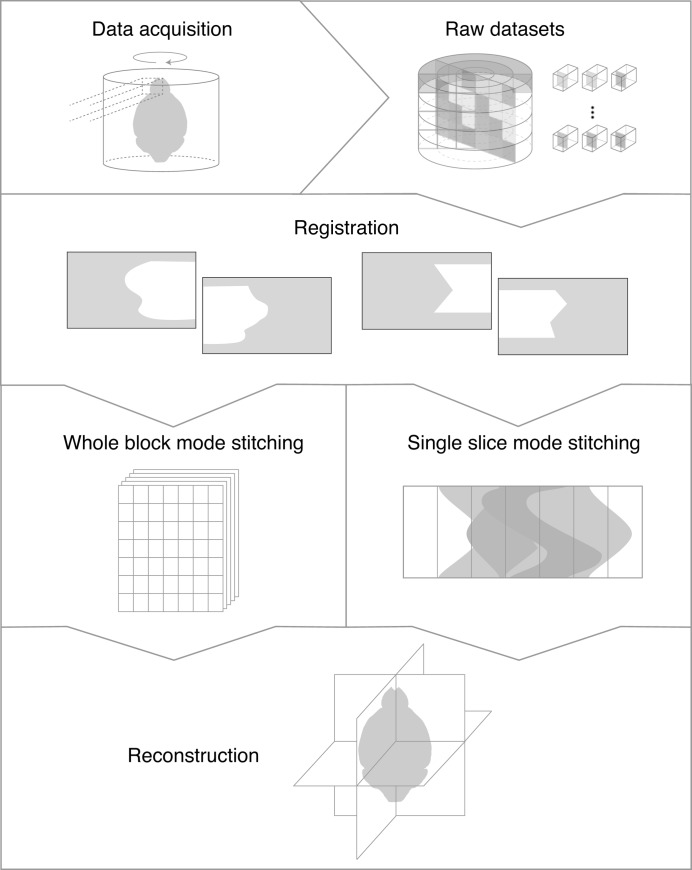
Overall workflow for *Tomosaic* data acquisition and reconstruction. Projections are obtained using sinogram-oriented acquisition (SOA) as shown on the right in Fig. 1 ▸. The alignment between subregions is then refined, after which the full three-dimensional dataset is assembled either by stitching together the sinograms as shown on the right in Fig. 1 ▸, or by collecting together full object projections as illustrated in Fig. 3 ▸ below. In either approach, one obtains the set of sinograms from all slices of the full object, and these object slice sinograms can then be fed to *TomoPy* (Gürsoy *et al.*, 2014 ▸) for parallelized reconstruction (Bicer *et al.*, 2016 ▸).

To implement the SOA approach, we have developed a software package for the processing of multi-field-of-view tomography data named *Tomosaic*. Because tomographic projections can be acquired at high speed at synchrotron light sources, one can obtain micrometre-resolution tomograms of centimetre-sized objects using millimetre-sized beams in a matter of hours using the *Tomosaic* approach. While image reconstruction from smaller datasets has already been demonstrated using a single workstations (Vescovi *et al.*, 2017 ▸), we extend here the reconstruction approach to work with teravoxel-sized reconstruction volumes and parallel computing. This is done *via* a message-passing-interface (MPI) enabled Python library which is written in a way that the same code can be run on standard workstations for smaller datasets, or on distributed clusters for data sizes that demand more memory and computing power. While the code is written in such a way that one can employ specific packages to read data in specific formats, and other packages for tomographic data reconstruction, the current version uses the *DataExchange* package for data file input/output (De Carlo *et al.*, 2014 ▸), and the *TomoPy* (Gürsoy *et al.*, 2014 ▸; Bicer *et al.*, 2016 ▸) and *Astra* (Pelt *et al.*, 2016 ▸) packages as the tomographic reconstruction backends. Moreover, in order to meet the demands of users with access to different levels of computational resources, *Tomosaic* provides two modes of reconstruction (WBM and SSM) as will be discussed further in §3.5. The overall workflow of *Tomosaic* is shown in Fig. 2 ▸, while our terminology for acquisition and reconstruction modes is summarized in Table 1 ▸.

## 2. Mosaic data acquisition   

**Figure 3:**
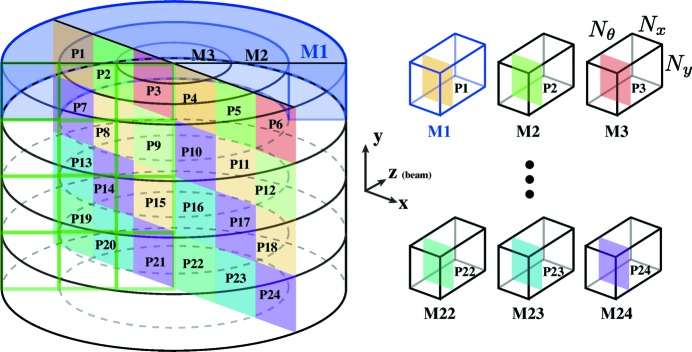
Schematic of the *Tomosaic* data collection approach, providing detail beyond Fig. 1 ▸. The rotation stage is first moved to projection position 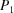, and the specimen is then rotated through 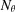 angular positions over a 180° range. This yields a mosaic ring projection dataset 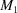. This is continued for all of the 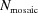 fields of view. In order to obtain a complete projection image at one rotation angle indexed by 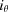, one must extract the corresponding projections from all of the mosaic ring datasets 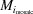 as shown on the right. In this example, the number of mosaic fields of view is 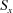 = 6 and 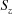 = 4, yielding 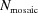 = 24.

The first step in our *Tomosaic* approach is to acquire the data, following the SOA approach shown in Fig. 1 ▸ and also Algorithm 3. This is shown in greater detail in Fig. 3 ▸. For projection position 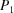, the rotation axis is shifted in 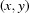 relative to the illumination/camera field of view, after which the first mosaic ring dataset 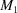 is acquired by rotating the specimen through 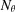 angular steps over a 180° range. This is then repeated for each of the 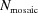 = 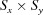 fields of view, so that the last projection position is 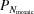 and the last mosaic ring dataset is 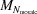. The actual acquisition also involves the collection of white-field (image with beam on and sample absent) and dark-field (image with beam off) data before acquiring sample projection data for each tile. These supply the needs of normalization correction, which are intended for the alleviation of beam intensity fluctuations, scintillator inhomogeneities, and thermally induced signal buildup in the CCD detector. Control of the beam shutters and sample stages are automated through a control script based on the Experimental Physics and Industrial Control System (EPICS). Our experience suggests that a robust tomography system should be equipped with not only stable and low-distortion optics and positioners but also reliable and properly optimized controlling hardware and software. The requirement is high particularly for experiments with a large number of rotation angles and short exposure times, since the speed of data saving can become the bottleneck in the loop and potentially lead to frame loss. High-speed storage media should be used as buffer zones for data transfer and writing, and the host console should have sufficient memory and multi-tasking capabilities in order to avoid the interruption of data I/O.

The *Tomosaic* pipeline works by reading in a file that names all of the grid positions involved in the dataset, so as to create the metadata necessary to merge and reconstruct the data. Each array (or ‘tile’) 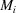 of mosaic projections is saved as a hierarchical data format (HDF) file, with a file naming scheme 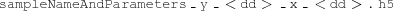, where 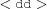 are two-digit integer indices counting from zero, identifying the grid position of the tile. Experimental metadata including the exposure time, beam current, motor readouts and the unique ID for each projection image are logged along with the tomogram data in the same file.

### 2.1. Experimental setup   

All data were acquired at the 32-ID beamline at the Advanced Photon Source. The setup consists of a 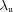 = 1.8 cm-period undulator operated at a low deflection parameter value of 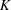 = 0.26, so that a single quasi-monochomatic peak at 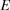 = 25 keV could be generated without loss due to crystal monochromators *etc*. For a sample at 68 m from the undulator, this produced a photon fluence rate of about 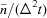 = 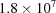
 photons s^−1^ µm^−2^, so that a specimen with an X-ray attenuation length of 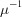 = 56.9 mm [an example value for poly(methyl methacrylate) or PMMA with 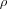 = 1.18 g cm^−3^] would receive a skin dose rate 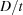 of 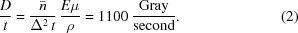
The sample was mounted on an air-bearing rotary stage PI-Micos UPR-160 AIR with motorized *x*–*y* translation stages located underneath and *x*–*y* piezo stages on top. Typical exposure times for a single projection image at one mosaic grid point and one rotation angle were 10–20 ms, and 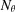 = 1500–6000 was used for the number of rotation angles at each grid point. Tomographic projections were recorded by using a 10 µm-thick LuAG:Ce scintillator to convert the propagation-enhanced X-ray intensity pattern into a visible-light image which was then magnified using a microscope objective onto a visible-light scientific CMOS camera (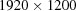 GS3-U3-23S6M-C for the charcoal sample, and 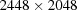 Point Gray GS3-U3-51S5M-C for larger specimens).

### 2.2. Data transfer and storage   

Data written on the experimental control computers at the beamline were transferred to the compute cluster Cooley, with a copy sent to the remote large-capacity data vault Petrel. Both facilities are developed and maintained by the Argonne Leadership Computing Facility (ALCF). Upon the completion of collection a rotation dataset at one tile position, an HDF5 file was created. This triggered *Ripple*, an event-driven data management software operating on an if-trigger-then-action basis (Chard *et al.*, 2017 ▸). File transfer to Cooley and Petrel was accomplished using *Globus* (Chard *et al.*, 2014 ▸), a data transfer and management service.

## 3. Mosaic data processing   

With the mosaic projection data acquired, we now describe the sequence of steps for data processing leading to a reconstructed three-dimensional image. The main steps were illustrated in Fig. 2 ▸.

### 3.1. Data downsampling   

To increase the speed of the pipeline and the data quality assessment, the user has the choice to reorganize the data into new folders containing binned versions of the original data. An *n*-fold binning is performed in the projection axis resulting in a 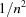 reduction in the raw data size and a 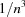 reduction in the reconstruction size. This approach also makes the pipeline more robust for finding the optimal solution for the metadata refinement, since each higher-resolution step can use the knowledge obtained from the lower-resolution (but more rapidly processed) step.

### 3.2. Registration of data   

With perfect translation and rotation stages, all of the 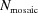 sinograms would be in perfect registration. In practice, this is not quite the case, so the commanded translations are used as a starting point for refinement of relative positions in a registration step. Because of the density of information in projections through thick objects, it is often difficult to recognize specific features and use feature-based alignment methods. Instead we follow the practice described previously (Vescovi *et al.*, 2017 ▸) and use the phase correlation method (Kuglin & Hines, 1975 ▸) which can efficiently determine sub-pixel registration through the use of matrix upsampling (Guizar-Sicairos *et al.*, 2008 ▸) as implemented in the Python library *Scikit-image*. The relative shift vector 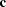 between two images of the same object is given by 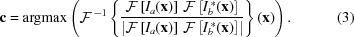
In other words, the relative shift vector is given by the coordinates of the global maximum in the Fourier cross-correlation map of images *a* and *b*. In principle, this can lead to four different possible shift vectors due to the periodicity of Fourier space (Preibisch *et al.*, 2009 ▸), but in our case we search for a maximum within a limited radius of zero shift because of the approximate correctness of the translation stage positions. Full-pixel shifts are handled in the obvious way, and sub-pixel shifts are implemented using the shift theorem of the Fourier transform as 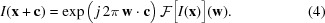
The results of registration are automatically exported as a text file with columns for the *y*-position and *x*-position of the tile, and the *y*- and *x*-axis shifts with regards to neighboring tiles to the right as well as the bottom.

### 3.3. Stitching and blending of data   

**Figure 4:**
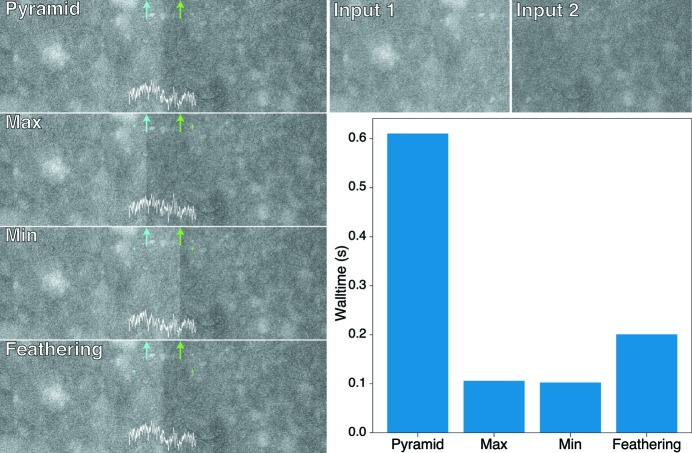
Blending results of two 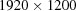 images given by pyramid blending, maximum blending, minimum blending and feather blending. The edges of the input images in the blending results are marked by blue and green arrows. In each subfigure in the left column, a white curve is shown to reflect the grayscale profile of the blended figure along its horizontal midline. A bar chart of the average wall clock time in ten runs of each algorithm is also provided.

Once the correct relative alignment of mosaic fields has been found, these fields must be stitched together. Slight errors in X-ray beam intensity normalization can lead to slight changes in apparent brightness at the boundary between one mosaic field and its neighbor; this can result in visible seams at mosaic field boundaries, which in turn cause ring artifacts in the tomographic reconstruction. Therefore, adjacent images must be blended through their overlapping region in order to result in a smooth transition. While we have compared several methods for doing this, pyramid blending (Adelson *et al.*, 1984 ▸) provides a good balance between computation speed and accuracy as shown in Fig. 4 ▸. The workflow of pyramid blending is shown in Algorithm 4. Briefly, the algorithm works by joining the image pair using a gradient mask at different down-sample levels. Through these operations, image features in multiple scales are captured and preserved. Background variations at lower spatial frequencies are blended more smoothly, while fine structures with higher spatial frequency content are given a quicker transition to prevent the ghosting effect. The benchmark testing shown in Fig. 4 ▸ demonstrates the performance of pyramid blending. The size of each input image is 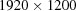, and the edge positions are marked by blue and green lines. For pyramid blending, no visible seams are found in the output image, in contrast to simple blending methods such as choosing the maximum or minimum value at each pixel in the overlap region. We also tried feather blending, which is essentially just a single Gaussian mask applied to the images at their original scales. Feather blending exhibits a ‘harder’ transition that leads to more ring artifacts in a tomographic reconstruction than one has when pyramid blending is used. The cost of pyramid blending is its relatively higher time consumption as compared with the other methods demonstrated here; however, the time needed for pyramid blending a full-resolution pair of images is still only about 0.6 s on an ordinary laptop computer, which is well acceptable.
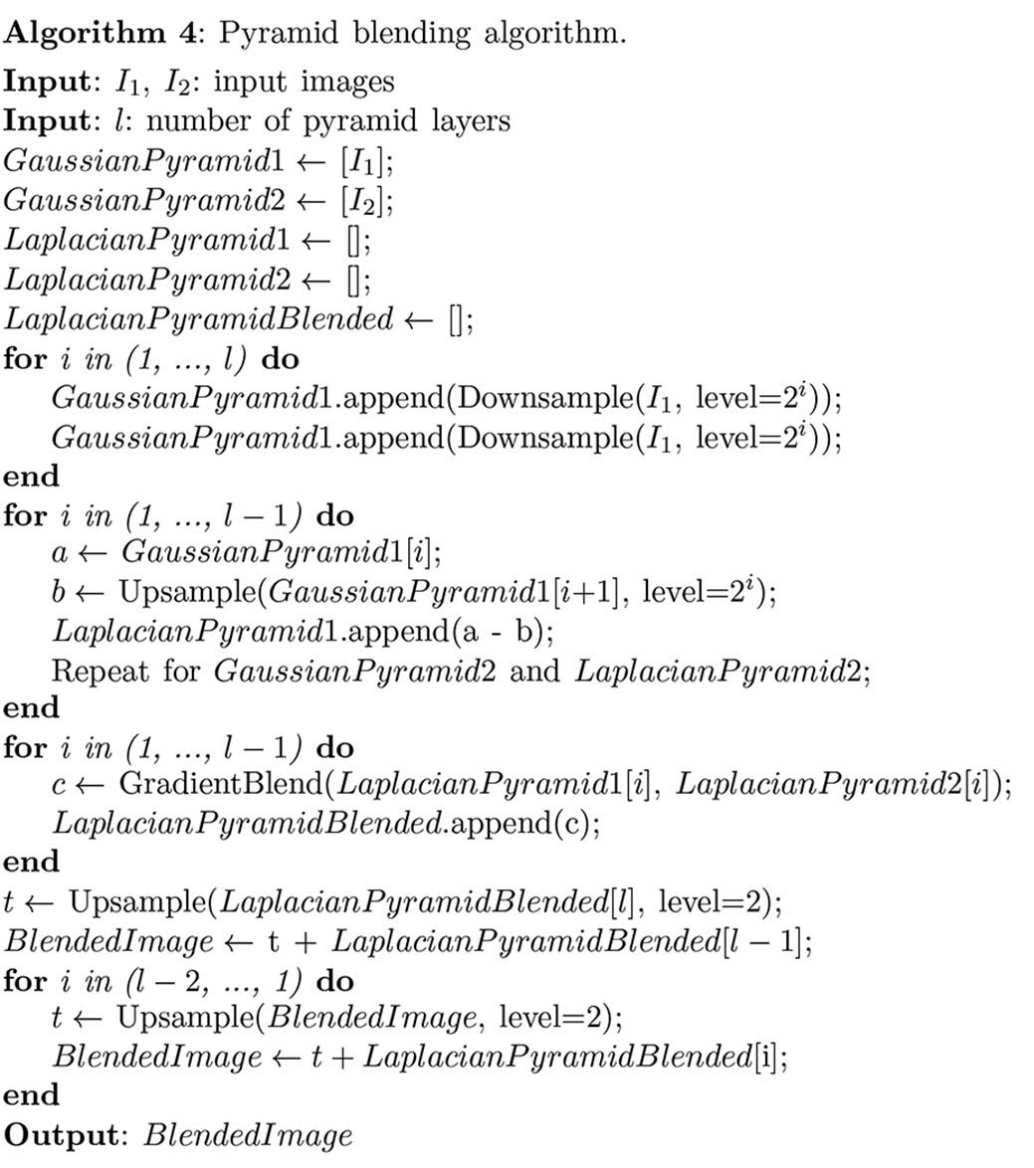


Using pyramid blending, *Tomosaic* reads in the shift data file created in the registration stage, and stitches the radiographs of all tiles in the grid into a panorama for each projection angle. The merged data are stored as a new HDF5 file. During stitching, projection images are normalized using flat fields and dark fields, so the stored data are in the form of floating points between 0 and 1.

### 3.4. Rotation center calibration   

Once the set of projection images has been assembled, the correct center of rotation must be found. When this is at a position different than what was assumed, objects that would be on the rotation axis appear to be rotating about it, leading to the appearance of arc artifacts in reconstructed images. Finding the correct center of rotation is a well known problem in tomography, and several approaches have shown varying degrees of success (Brunetti & De Carlo, 2004 ▸; Donath *et al.*, 2006 ▸; Vo *et al.*, 2014 ▸; Yang *et al.*, 2017 ▸).

**Figure 5:**
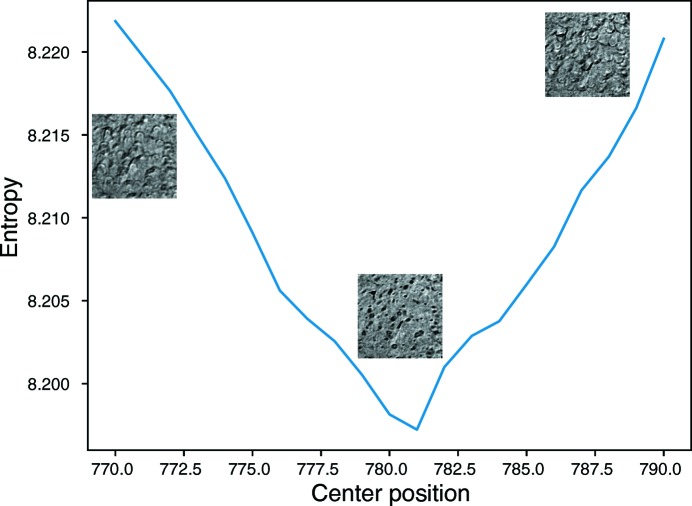
Identification of the correct center of rotation in a tomographic slice reconstruction by using image entropy of reconstructed images [equation (5)] as a metric (Donath *et al.*, 2006 ▸). In this case, the center of rotation turned out to be at pixel index 781; a range of pixel centers from 770 to 790 are shown.

Artifacts represented by the U-shape distortion of point features can seriously deteriorate image quality if the center is not correctly set. *Tomosaic* uses an entropy-based optimization approach (Donath *et al.*, 2006 ▸) for finding the rotation center for every row in the tile grid. The concept of image entropy is defined as 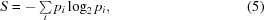
where 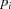 is the probability of a certain grayscale value *i*. It has been observed that a reconstruction image with an incorrect center setting has a wider distribution of grayscale values due to the smearing effects of the artifacts, and is associated with a higher entropy. Thus, the correct center value 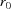 can be found through 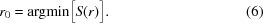
A demonstration of the entropy-based center-searching algorithm is shown in Fig. 5 ▸. A scan from 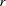 = 770 to 790 was carried out, and the correct center position of 781 was successfully identified by the sharp minimum of the curve.[Chem scheme5] 
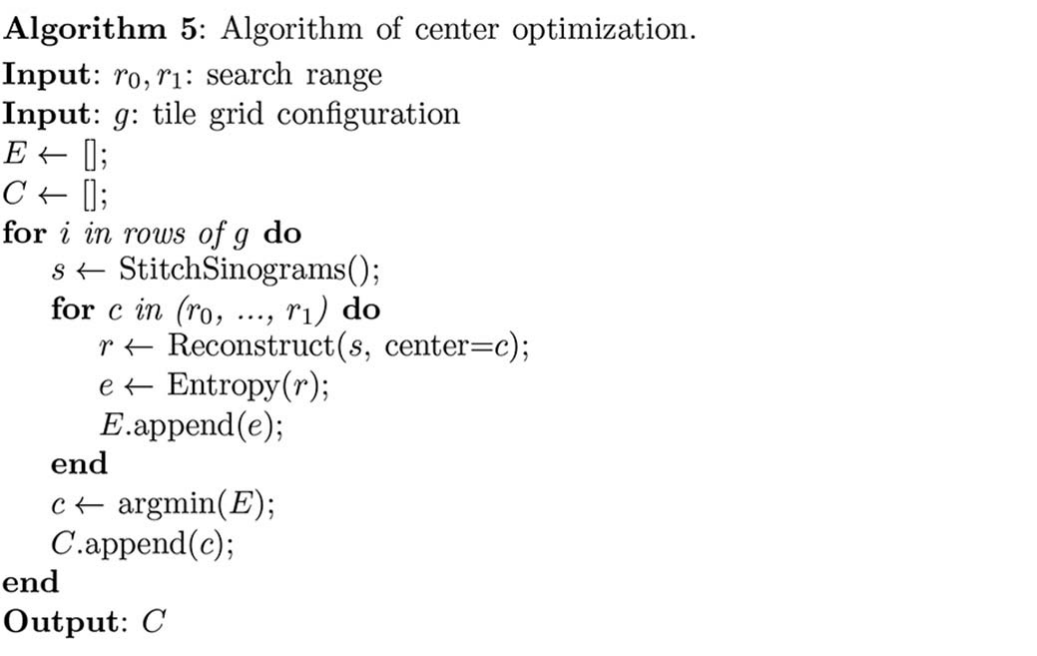


*Tomosaic* calculates the rotation center for each row in the tile grid. The results are exported as a text file listing the row number and the corresponding center position. Also, reconstruction images for the range of center positions searched are kept in the hard drive as well, so that one could manually re-examine the images if the automated outcomes are not satisfactory.

### 3.5. Reconstruction   

In order to flexibly meet the needs of users and to function properly on various platforms with different hardware conditions, *Tomosaic* provides two modes for tomographic reconstruction of the three-dimensional object from the rotation sets of two-dimensional projection images as noted in Table 1 ▸:

(i) Whole-block mode (WBM). In this mode, a single merged dataset containing aligned and stitched projections for all rotation angles as described in §3.3 is fed to the tomographic reconstruction package. In practice, the dataset is stored on the hard drive, and data are read as required. Algorithm 6 shows the workflow of this mode. This mode is able to deal with sub-pixel registration shifts transverse to and along the rotation axis, or 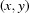 shifts of each mosaic field of view. Because each projection slice along the rotation axis direction can be reconstructed independently of all other projection slices, this task is trivially parallelizable on multi-node clusters. Since the stitching of projections is done in both the 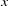
- and 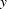-direction, smooth blending and sub-pixel registration in both axes can be preserved.
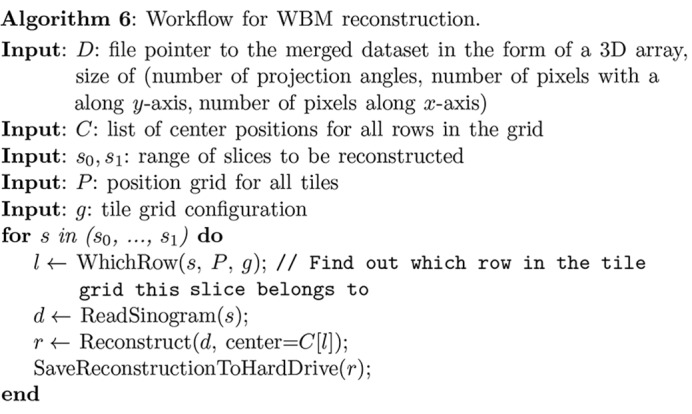


(ii) Single-slice mode (SSM). In this mode, sinograms rather than projections are stitched using Algorithm 7. In this case, one can register mosaic sinograms at full-pixel precision in the direction of the axis of rotation, with further sub-pixel precision achievable in the transverse direction only. The advantage that one gains in return is that SSM demands far less storage space and time, because the stitching of projections for all angles is not needed for reconstruction. As a result, reconstruction can be done on personal workstations or even laptop computers in this way. Also, because this mode provides an *ad hoc* approach to reconstruct a small number of slices, it is a convenient solution for delivering a fast preview of the data quality.
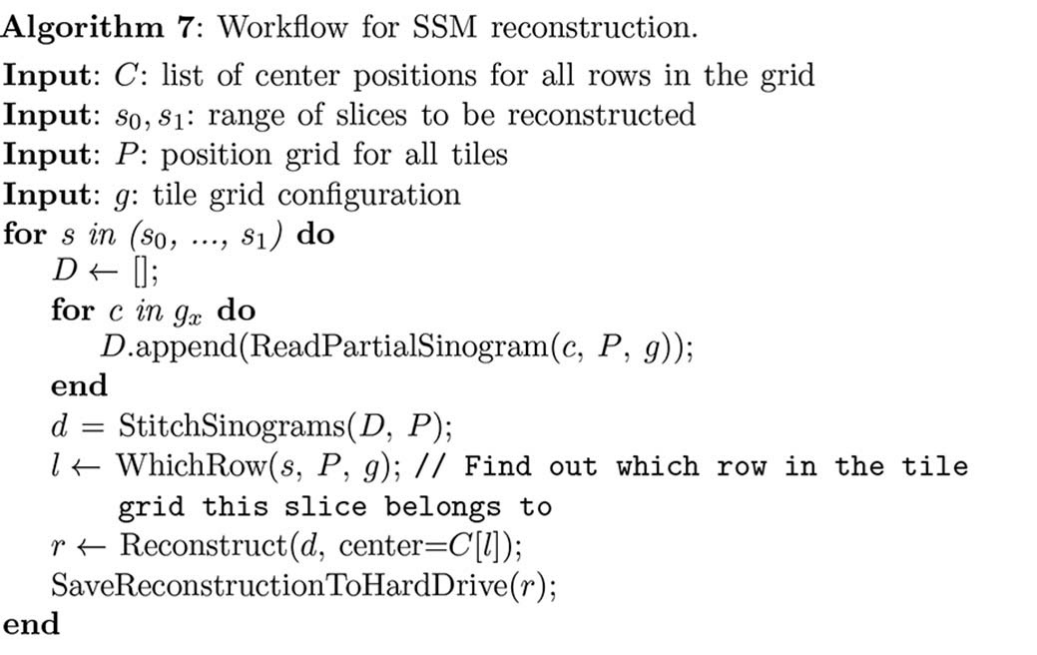


In both cases we assume that the registration between subfields has already been determined as described in §3.2. For both modes, *Tomosaic* uses the package *TomoPy* (Gürsoy *et al.*, 2014 ▸) for volumetric image reconstuction using one of several available standard algorithms, including on distributed computers (Bicer *et al.*, 2016 ▸). This package implements a transport-of-intensity-based approach (Paganin *et al.*, 2002 ▸) for the reconstruction of phase contrast features from the projections acquired at a distance from the sample. Because the ‘gridrec’ implementation (Dowd *et al.*, 1999 ▸) of filtered backprojection offers rapid non-iterative reconstructions, we use it to minimize computing time when working with the very large data sets described here.

**Figure 6:**
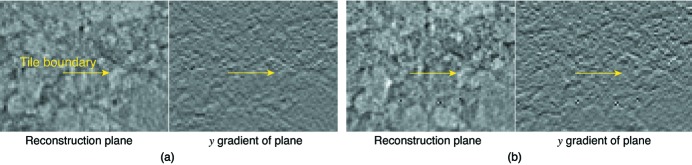
A comparison of reconstruction quality between the (*a*) single-slice (SSM) and (*b*) whole-block (WBM) modes of *Tomosaic* reconstructions. These images are of an *x*–*y* reconstruction plane from a the charcoal sample described in §4, where *y* is in the vertical direction (the direction of the axis of rotation in our geometry with a horizontal illuminating beam from a synchrotron). The *x*–*y* plane view is at the intersection between two tiled projection datasets, one above the other (see Fig. 3 ▸); the *y* gradient image is also shown next to the reconstruction plane view. Because the SSM reconstruction does not perform sub-pixel alignment or blending between object slices in the vertical direction (so as to fit within the memory and computing power constraints of a single workstation), one can see a discontinuity at the tile intersection as indicated by an arrow. This discontinuity is removed in the WBM reconstruction, which includes aligment and blending in the vertical direction (thus demanding more memory and computing power, making it better suited for use on a parallelizable computing cluster).

In order to compare reconstruction quality between WBM *versus* SSM reconstructions, in Fig. 6 ▸ we compare reconstructions by looking at the reconstruction quality along the vertical direction. The SSM reconstruction shows a discontinuity at the border between two data acquisition tiles (Fig. 3 ▸), while the WBM does not. This illustrates how the single-workstation-compatible SSM approach gives a quick and useful view of the data by only considering one object slice at a time, but the full dataset registration and blending capabilites of the WBM approach (which is best done on a larger compute cluster) are required for obtaining the highest reconstructed image quality.

### 3.6. Scalability   

**Figure 7:**
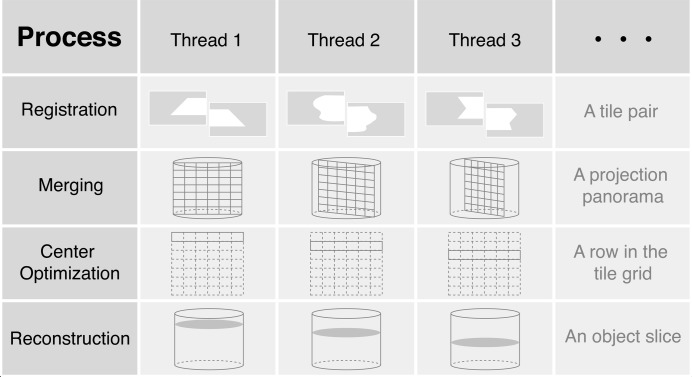
Pattern of thread allocation for registration, merging, center optimization and reconstruction.

Because the WBM reconstruction approach can be applied to datasets that are too large to fit within the memory of most single-node computers, and also to gain speed in data processing, the WBM steps have been parallelized using the open-source message passing interface package *MPI for Python* (Dalcín *et al.*, 2005 ▸, 2008 ▸, 2011 ▸). Job allocation among available ‘ranks’ or processing nodes is done automatically within *Tomosaic*, so that the only input needed from a user is the number of ranks to use. The pattern of thread allocation varies adaptively for different stages of the *Tomosaic* workflow. Fig. 7 ▸ illustrates the parallelization mechanism for registration, merging (*i.e.* stitching and blending), center calibration and reconstruction.

Test runs of parallel processing have been performed on laptops, workstations and the multi-node supercomputer Cooley located at the Argonne Leadership Computing Facility. Benchmark data will be presented in §4.

## 4. Results   

*Tomosaic* has been applied to a three-dimensional imaging and processing of a collection of samples. In this section, we describe its use for imaging two samples using 25 keV monochromatic X-rays as described above, with data processing times listed in Table 2 ▸.

**Figure 8:**
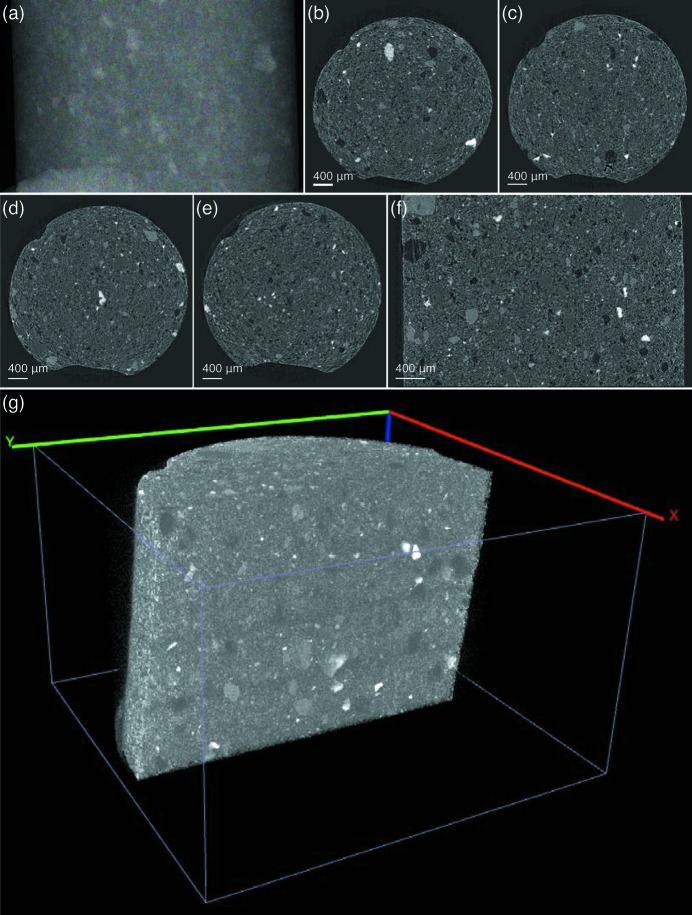
Tomosaic reconstruction of a charcoal specimen. (*a*) One of the panorama projection images obtained using pyramid blending. (*b*)–(*e*) Selected reconstruction slices in the *x*–*z* plane. The positions of these slices along the *y*-axis are indicated by the dashed lines in (*b*). (*f*) Cross sections of the entire reconstructed volume in the *x*–*y* plane. (*g*) Three-dimensional rendering of the reconstructed volume, truncated vertically to reveal internal structure.

The first dataset is of an activated charcoal pellet with an approximate diameter of 4 mm. For each scan, 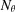 = 4500 rotation angles were uniformly sampled in the 0–180° interval. Using a 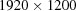 pixel camera, a 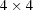 tile grid was used to cover the entire sample, resulting in a total data size of 302 GB (of 16-bit unsigned integers). The complete reconstructed volume has a voxel dimension of 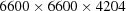 with a pixel size of 0.6 µm. The processing of this dataset was performed on a workstation equipped with dual Intel E5-2690V2 CPUs (ten cores each, at 3 GHz) and 128 GB physical memory. Parallelized with 20 threads, registration of the 16 tiles was completed in just 25 s. With pyramid blending and 20 threads, the stitching of all projections was finished within 176 min. One of the stitched projection images is shown in Fig. 8(*a*) ▸. Reconstruction of the full dataset was then performed in 500 min, also using 20 threads. Figs. 8(*b*)–8(*f*) ▸ show cross sections cut along the *x*–*y* and *x*–*z* planes. No ring artifacts are found in the horizontal slices. The full reconstruction volume was rendered using *Vaa3D* (Peng *et al.*, 2010 ▸) yielding the visualization shown in Fig. 8(*g*) ▸. The volume is truncated vertically to reveal the internal structure. This dataset, as well as the single data file merged using *Tomosaic*, has been made available on TomoBank, a public repository of tomographic datasets and phantoms (De Carlo *et al.*, 2018 ▸), with a sample ID of 00078.

We also used *Tomosaic* to image a metal-stained epoxy-embedded mouse brain specimen (10.7 mm × 9.2 mm × 13.2 mm, images of which will be published separately) which involved more challenges due to its significantly larger volume. The final pixel size of the acquisition was 0.8 µm. In order to illuminate the whole sample, a 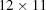 tile of partial tomograms with 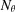 = 4500 angles was used. The projection images were stored as 16-bit unsigned integers, yielding a total data size of 5.8 TB. The full-resolution reconstruction of the dataset was conducted using the compute cluster Cooley at Argonne, which has 126 computation nodes, each possessing two Intel E5-2620 v3 processors (12 cores in total) and 384 GB RAM. Data registration was conducted using 100 threads (20 nodes with five threads per node) and was performed within 30 s. Stitching and blending took approximately 10 h with 250 threads. The final reconstruction was carried out with 100 threads (50 nodes with two threads per node) in about 50 h. After the entire process, we obtain the volume mapping of the entire sample containing 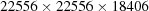 = 9.36 × 10^12^ voxels (or 37.4 TBytes at 32-bit depth) as indicated in Table 2 ▸.

## 5. Discussion   

As illustrated here, increases in the tomographic field of view to the teravoxel scale and beyond dictate the development of data acquisition, management and reconstruction pipelines so as to keep experiments within the bounds of what is computationally feasible. In this paper we introduced a pipeline for mosaic tomography. Considering the strong similarities among various tomographic techniques, we designed *Tomosaic* using a modular strategy so that its functionality can be made available for other techniques (for example, for image registration and merging).

The use of high-performance computing (HPC) systems for the solution of large-scale tomography problems is becoming more prevelant. While software routines that enable the management (Li *et al.*, 2017 ▸) and visualization (Ahrens *et al.*, 2005 ▸) of petabyte-scale data on HPCs have been made available to the community, they mostly work with gigavoxel-scale three-dimensional image data that were already acquired, assembled and reconstructed. We have seen fewer examples of software packages that fully exploit the potential of HPCs for the upstream processing of tomography data, such as the acquisition of three-dimensional volumes from raw projections or their alignment. The major issue lying between most existing beyond-field-of-view tomographic reconstruction routines and their HPC deployment is either the lack of an interface to distribute jobs among multiple computational nodes, or deficiencies in the level of automation across the entire tomographic processing pipeline. Our current implementation of *Tomosaic* has an abstraction layer on top of the Python bindings in order to make the whole pipeline suitable for HPC systems. This modularity and layering allows one to access the full range of capabilities and features of the toolbox (such as pre-processing functions) using different computing resources.

Since *Tomosaic* is available as an open-source project, it is important that the package can handle data generated from a wide range of light sources across the world, where the data format usually varies from case to case. Therefore, a universal data reader and converter is needed as the I/O backend for *Tomosaic*. A published scheme, *DataExchange*, has the potential to serve as the bridge between stored raw data and the *Tomosaic* pipeline (De Carlo *et al.*, 2014 ▸). With the *DataExchange* module, one can import data from a range of synchrotron facilities worldwide, all of which have their unique format for storing experimental data. The conversion of tomographic data from these various facilities into the *DataExchange* format makes it easier for *Tomosaic* to potentially benefit a wide range of users in the imaging community.

For the future, one of the largest problems for which improved solutions are sought involves tile alignment and assembly. The phase correlation method currently being used is not always reliable for ultra-thick specimens due to the shortage of high-contrast features, and is vulnerable to noise. Artificial fiducial marks added to the specimen may improve the reliability of correlation registration for thick samples. We can also consider application of an iterative reprojection approach [sometimes called a bootstrapping approach (Dengler, 1989 ▸), for which speedups are available when using iterative tomogram reconstruction methods (Gürsoy *et al.*, 2017 ▸)] though this will be challenging given large datasizes. We may also apply convolutional neural network-based classifiers as an automated gage for the quality of registration outcome.

## 6. Conclusion   

This paper describes the modules currently available in the *Tomosaic* framework. The most important part of the pipeline is it scalability that permits tomography experiments to have their field of view extended as much as necessary. The *Tomosaic* code produced thus far is publicly available as a package of the same name on GitHub.

## Figures and Tables

**Table 1:** Terminology used in this work for data acquisition and reconstruction approaches

Local tomography acquisition (LTA)	A data acquisition mode where one collects tomography data from local regions of the sample, and stitches together the individual reconstructions.
Projection-oriented acquisition (POA)	A data acquisition mode where one collects a full panoramic projection image at each rotation angle.
Sinogram-oriented acquisition (SOA)	A data acquisition mode where one collects 180° projections at each position on the sample. This is the method used in *Tomosaic.*
Whole-block mode (WBM)	A reconstruction mode in *Tomosaic* where projections are stitched for each angle, after which reconstruction is performed on the merged dataset.
Single-slice mode (SSM)	A reconstruction mode in *Tomosaic* where sinograms for a slice are extracted and stitched, after which this particular object slice is reconstructed.

**Table 2:** Scalability of *Tomosaic* data processing with data set size and number of nodes

Sample	Charcoal	Mouse brain
Pixels per tile	1920 × 1200	2448 × 2048
Number of rotations *N*_θ_	4500	4500
Pixel size (µm)	0.6	0.8
Mosaic tile grid size	4 × 4	12 × 11
Full recorded data volume (TB)	0.30	5.8
Threads for registration	20	100
Time for registration (s)	25	30
Threads for merging	20	250
Time for merging (h)	2.9	10
Threads for center calibration	4	11
Time for center calibration (h)	0.1	0.25
Threads for reconstruction	20	100
Reconstructed volume voxels	6600 × 6600 × 4204	22556 × 22556 × 18406
Reconstructed data volume (TB)	0.73 (32 bit)	37.4 (32 bit)
Time for reconstruction (h)	8.3	50
